# Endpoints for Lymphatic Filariasis Programs

**DOI:** 10.3201/eid1304.061063

**Published:** 2007-04

**Authors:** Caroline A. Grady, Madsen Beau de Rochars, Abdel N. Direny, Jean Nicolas Orelus, Joyanna Wendt, Jeanne Radday, Els Mathieu, Jacquelin M. Roberts, Thomas G. Streit, David G. Addiss, Patrick J. Lammie

**Affiliations:** * Centers for Disease Control and Prevention, Atlanta, Georgia, USA; † Hopital Ste. Croix, Leogane, Haiti; ‡ University of Notre Dame, Notre Dame, Indiana, USA

**Keywords:** lymphatic filariasis, mass drug administration, elimination, microfilaremia, Wuchereria bancrofti, diethylcarbamazine (DEC), albendazole, dispatch

## Abstract

In 2000, annual mass administration of diethlycarbamazine and albendazole began in Leogane Commune, Haiti, to interrupt transmission of lymphatic filariasis (LF). After 5 years of treatment, microfilaremia, antigenemia, and mosquito infection rates were significantly reduced, but LF transmission was not interrupted. These finding have implications for other LF elimination programs.

Globally, more than 1 billion persons are at risk for lymphatic filariasis (LF), a mosquito-transmitted parasitic infection that causes lymphedema and hydrocele. Transmission of LF can be interrupted by annual mass treatment with drugs that target microfilariae, the stage of the parasite that circulates in the blood (*1*,*2*). Programs in Egypt, Samoa, Zanzibar, and other settings have recently completed 5 rounds of mass drug administration (MDA) (*3*,*4*), a proposed endpoint for treatment, and are now confronted with a critical question: can MDA be stopped without fear of recrudescence of LF infection?

The LF demonstration project in Leogane, Haiti, was designed as an operational research project to monitor the impact of MDA on LF infection in a high-prevalence setting. The intervention was annual MDA with diethylcarbamazine (DEC) and albendazole. We discuss how sentinel site data were used to determine whether to continue mass MDA after 5 rounds and how our experience may be relevant to other LF programs.

Leogane is located 30 km west of Port-au-Prince. Before the first MDA in 2000, 4 sentinel sites in Leogane commune were selected for annual follow-up of microfilaremia and antigenemia (*5*). Monitoring of filarial infection prevalence in the vector, *Culex quinquefasciatus,* began in these sites 3 months before the first MDA, using CDC gravid traps (Model 1712, J.W. Hock Co., Gainesville, FL, USA) (*6*); testing continued on a semimonthly basis. Infected mosquitoes were defined as those carrying microfilariae or larvae (L1–L3); L3 were the infectious larval stage. Protocols for collecting data from sentinel sites were approved by the Centers for Disease Control and Prevention Institutional Review Board and the Ethics Committee at Hopital Ste. Croix.

In October 2000 and every October thereafter, DEC (6 mg/kg) and albendazole (400 mg; GlaxoSmithKline, Brentford**,** UK) were co-administered at stationary posts to persons >2 years of age, excluding pregnant women and persons too ill to receive the drugs. Adverse events were monitored each year by recording the number of persons who returned to distribution posts with complaints. Cluster surveys were conducted after the first and third MDA to assess coverage and the effect of health messages on compliance (*7*,*8*).

Reported coverage in 2000–2004 for MDAs 1–5 was 69%, 50%, 84%, 89%, and 104%, respectively (Table). Decreased coverage in 2001 may have been related to a relatively high incidence of adverse events caused by death of microfilariae and adult worms during the first MDA (*9*). The increase in reported coverage in 2004 may have been due to an influx of displaced persons from areas of Haiti affected by civil strife. Survey-based coverage in 2000 and 2002 was 71% and 79%, respectively (*7*,*8*). Adverse events diminished with each year of treatment, from 23.1% of persons treated during 2000 to 3% during 2004 (p<0.0001).

**Table:** Drug coverage for Leogane Commune, Haiti

	2000	2001	2002	2003	2004
No. treated*	74,000	55,000	94,000	102,000	122,000
Reported coverage†	69%	50%	84%	89%	104%
Surveyed coverage‡	71%	NA	79%	NA	NA
Adverse events§	23%	16%	9%	8%	3%

*Based on tally counts from distribution posts. †Using 108,000 as the total population of Leogane determined by cluster survey in 2000, adjusted for annual population growth. International Data Base (IDB): available from http://www.census.gov/ipc/www/idbprint.html ‡Published data (*7**,**8*) §Based on the number of persons who reported to distribution posts with reportts adverse events as a percentage of the reported coverage. See reference (*9*) for a more detailed discussion of adverse events associated with the first round of mass drug administration..

Baseline microfilaremia prevalence rates were 0.8%, 7%, 12%, and 16% in the sentinel sites of Mapou, Barrier-Jeudi, Masson-Mathieu, and Leogane, respectively (Figure 1). Microfilaremia prevalence decreased significantly in each of the sentinel sites (Mapou, p = 0.0291; each of the other sites, p<0.0001). Antigenemia prevalence declined less dramatically, by 18.6%, 34.6%, 74.2%, and 54.7% in Mapou, Barrier Jeudi, Masson-Mathieu, and Leogane, respectively (p<0.0001 in all sites except Mapou).

**Figure 1:**
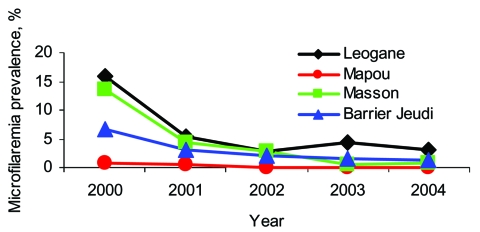
Microfilaremia prevalence determined by screening thick blood films before and 6–9 months after annual mass drug administration in sentinel sites in Leogane Commune, Haiti. Blood samples (20 μL) were collected from 7–9 PM.

Baseline mosquito infection rates 3 months before the first MDA were 0.5%, 2.9%, 3.5%, and 4.0% in Mapou, Masson-Mathieu, Leogane, and Barrier-Jeudi, respectively. After MDA 1, infection prevalence decreased significantly only in Masson-Mathieu (p = 0.004); however, after 2 rounds of MDA, infection was reduced significantly at all sites (p<0.007) except Mapou (Figure 2). After MDA 4, infection prevalence was 0% during some months at all sentinel sites, although infected mosquitoes were detected sporadically at all sites but Mapou. The prevalence of infective mosquitoes was lower than the prevalence of infected mosquitoes (p<0.05), but parallel declines were observed after MDA (data not shown).

**Figure 2:**
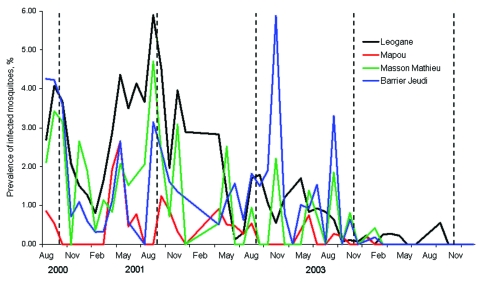
Prevalence of infection (microfilaremia, L1, L2, and L3) in dissected mosquitoes collected by using gravid traps in sentinel sites in Leogane Commune, Haiti. Data are aggregated on a monthly basis. Dashed lines represent annual mass drug administration intervention.

These data were collected to monitor progress and to provide a basis for programmatic decisions. In January 2005, 3 months after MDA 5, a meeting was convened in Leogane with program and ministry staff to discuss the need for further MDA. Sustained reductions in LF infection in both humans and mosquitoes demonstrated the substantial effects of the intervention through 4 MDA rounds. However, persistent antigenemia and sporadic parasitemia were detected at all sentinel sites. Project leaders adopted a conservative approach and planned for MDA 6 on the basis of the evidence of continued transmission in the sentinel sites.

Several factors supported this decision, including detection of infections in both humans and mosquitoes and concerns about systematic noncompliance (*8*), the potential for inflated coverage estimates due to population migration, the nonrepresentativeness of sentinel sites for estimating MDA impact, and the insensitivity of blood smears for monitoring microfilaremia. Since the cost of an additional MDA was not prohibitive, project staff decided that the evidence of continued transmission, the health benefits of mass treatment to the community, and the risk of stopping treatment prematurely justified a sixth round of MDA in October 2005. Results from Leogane and other programs have demonstrated that 5 rounds of MDA may not be sufficient to interrupt transmission when baseline antigenemia is high, whereas in low-prevalence areas <5 rounds appears to be adequate (*3*). Mathematical models as well as program experience suggest that the number of MDAs required depends on baseline intensity of infection, assuming adequate coverage (*10*,*11*).

Adequate monitoring data are important for making decisions regarding continuation of mass treatment. Microfilaremia and ICT testing are the gold standards for measuring the impact of MDA; however, nocturnal blood collection required for microfilaria testing is inconvenient, the high cost of the ICT is a concern (≈$2.65), and the sensitivity of both tests decreases as LF intensity and prevalence decline.

Entomologic monitoring provides an alternative method of measuring the impact of MDA on transmission. Although it circumvents the human cost of repeated blood collection and provides a direct, real-time measure of potential transmission, continuous mosquito collection and dissection were more costly and labor-intensive than other monitoring methods that we used. Conducting intermittent rather than continuous collections may be an alternative approach.

The limitations of these monitoring tools highlight the need for more sensitive, standardized tools to help programs define MDA endpoints and to conduct surveillance. Antibody responses may develop before patent infection and serve as a cumulative measure of filarial exposure and a proxy for transmission (*12*). In Egypt, antibody surveys of children beginning school were used to monitor for incident exposure, indicative of ongoing transmission (*3*). Additional studies are needed to validate antibody tests and to analyze the relationship between antibody prevalence and transmission intensity.

In summary, MDA-based LF programs, including that in Leogane, lead to dramatic declines in filarial infection in humans and mosquitoes after several annual rounds of MDA (*2*,*3*,*13*–*15*). The outcomes of the Leogane project and others that have completed 5 rounds of MDA strongly suggest that the duration of treatment is related to the baseline transmission intensity and infection prevalence. Several issues—population migration, systematic noncompliance, and sentinel site bias—have emerged as variables that complicate decision making. Investigating their effect on infection and transmission in an operational context is critical.
